# Instruments to Assess Secondhand Smoke Exposure in Large Cohorts of Never Smokers: The Smoke Scales

**DOI:** 10.1371/journal.pone.0085809

**Published:** 2014-01-21

**Authors:** Maria Misailidi, Manolis N. Tzatzarakis, Mathaios P. Kavvalakis, Yiannis Koutedakis, Aristidis M. Tsatsakis, Andreas D. Flouris

**Affiliations:** 1 FAME Laboratory, Centre for Research and Technology Hellas, Trikala, Greece; 2 Department of Exercise Sciences, University of Thessaly, Trikala, Greece; 3 Regional Directorate of Primary and Secondary Education of Western Greece, Patras, Greece; 4 Centre of Toxicology Science and Research, Medical School, University of Crete, Iraklio, Greece; 5 School of Sports, Performing Arts and Leisure, University of Wolverhampton, Walsall, United Kingdom; Kyushu University Faculty of Medical Science, Japan

## Abstract

The objectives of this study were to: (i) to develop questionnaires that can identify never-smoking children and adults experiencing increased exposure to secondhand smoke (SHS+), (ii) to determine their validity against hair nicotine, and (iii) assess their reliability. A sample of 191 children (85 males; 106 females; 7–18 years) and 95 adult (23 males; 72 females; 18–62 years) never-smokers consented to hair nicotine analysis and answered a large number of questions assessing all sources of SHS. A randomly-selected 30% answered the questions again after 20–30 days. Prevalence of SHS+ in children and adults was 0.52±0.07 and 0.67±0.10, respectively (p<0.05). The Smoke Scale for Children (SS-C) and the Smoke Scale for Adults (SS-A) were developed via factor analysis and included nine questions each. Positivity criteria for SS-C and SS-A via receiver operating characteristics curve analysis were identified at >16.5 and >16, respectively. Significant Kappa agreement (p<0.05) was confirmed when comparing the SS-C and SS-A to hair nicotine concentration. Reliability analyses demonstrated that the SS-C and SS-A scores obtained on two different days are highly correlated (p<0.001) and not significantly different (p>0.05). Area under the curve and McNemar's Chi-square showed no pair-wise differences in sensitivity and specificity at the cutoff point between the two different days for SS-C and SS-A (p>0.05). We conclude that the SS-C and the SS-A represent valid, reliable, practical, and inexpensive instruments to identify children and adult never-smokers exposed to increased SHS. Future research should aim to further increase the validity of the two questionnaires.

## Introduction

Despite a multitude of anti-smoking campaigns being active worldwide, the number of smokers is currently larger than at any other time in human history [1], [2]. As a consequence, secondhand smoke (SHS) remains a major threat to public health [1], [3] due to its adverse health effects in both children [4], [5] and adults [2], [6]. While it was previously believed that only daily SHS exposure for years (e.g., living with a smoker) can influence health, recent evidence has shown that even brief SHS exposures can contribute to disease pathogenesis [7]–[9]. Thus, it is now unanimously acknowledged that there is no safe level of exposure to SHS [10], [11].

An essential first step towards minimizing the health effects of SHS is to accurately assess the exposure level of individuals [12]. To date, this has been accomplished primarily through questionnaires [13]–[15], and to a lesser extent by measuring biomarkers (e.g., nicotine) in body fluids [16]–[18] and secondary measurement [19], [20]. The popularity of questionnaires stems from their practicality and low cost, especially when testing large cohorts [12], [21]. Despite these advantages, most of the existing questionnaires aiming to assess SHS exposure have not been validated against a referenced standard [12]. For those questionnaires that have undergone validation, the reference standard adopted was either nicotine or cotinine levels in saliva [22]–[24], blood [25]–[27], or urine [28]–[30]. However, given their short half-life, these biomarkers can provide information on SHS exposure only for the preceding 48–72 hours [31]. This is a crucial limitation since the reference standard used to validate a SHS questionnaire should be able to reflect individual exposure to SHS for periods longer than a few days. The validity of hair nicotine to detect varying levels of SHS exposure has been repeatedly demonstrated [32]–[34]. For instance, we recently demonstrated that hair nicotine concentration is an effective method for assessing SHS exposure in both children and adults for the preceding three months [35]. As average monthly hair growth is 1 cm, a 3-cm hair sample can be used to assess SHS exposure during the past three months [34]–[39] since nicotine is incorporated into the hair shaft throughout hair life [40], [41]. In this light, the purpose of this study was threefold: (i) to develop questionnaires that can identify children and adult never-smokers experiencing increased SHS exposure, (ii) to determine their validity against hair nicotine, and (iii) assess their reliability.

## Methods

### Ethics Statement

The study was conducted according to the principles expressed in the Declaration of Helsinki and was approved by the University of Thessaly Ethics Review Board (protocol no. 201) as well as the Regional Directorate of Primary and Secondary Education of Western Greece.

### Participants

Particular attention was given during the recruitment procedures to ensure that the participants represented socioeconomic, ethnic, and urban or rural groups of never-smokers as they occur in the Greek population. Specifically, measurements were conducted in the city of Patras (214,000 inhabitants; Greece's 3^rd^ largest urban area) and the surrounding areas. The locations selected for data collection represented equally urban and rural areas, as well as a variety of socioeconomic and ethnic groups as they exist in Greece. Children participants were recruited at four elementary and two high schools located in Patras and in its surrounding areas. All 280 students of these schools were invited to participate in the study, and 191 children (106 females; 85 males; age range 7–18 years) agreed. Adult participants were recruited at the city center, as well as at suburban and rural areas surrounding the city. A total of 130 adults were invited to participate, and 95 of them (72 females; 23 males; age range 18–62 years) agreed. Written informed consent was obtained from all 286 volunteers (i.e., 191 children and 95 adults) and their parents (for children) after full oral and written explanation of the experimental procedures. The only exclusion criterion adopted in the study was current or previous smoking.

### Collection Period and SHS-related Legislation

We used a relatively short data collection period to eliminate the effect of altering seasons on SHS exposure characteristics. All data were collected during February and March 2010. In Greece, this represents the end of the winter period, when people still spend a major part of their time indoors as environmental temperatures remain relatively low. The SHS-related legislation in Greece at the time the data was collected was quite lenient and largely ineffective offering only partial protection (i.e., non-smoking areas) to nonsmokers in public settings. At the same period, Australia, Canada, Ireland, New Zealand, Singapore, South Africa, UK, and USA had a total smoking ban in all workplaces and public places. In Finland, France, Italy, Latvia, Malta, Sweden, Slovenia, and The Netherlands, smoking in public places was allowed only in separate smoking rooms. The other EU countries (including Greece) offered a partial protection to nonsmokers in public settings [42] while no SHS-related legislation was actively enforced in the remaining countries in the world. Therefore, the SHS exposure conditions for nonsmokers in Greece at the time the data were collected represents very effectively those conditions faced by the vast majority of nonsmokers across the world.

### Experimental Protocol

The data were collected in two stages. During the first stage, participants completed the questionnaires which included a total of 51 questions for children and 67 questions for adults pertaining to all relevant sources of SHS exposure (i.e., home, occupational, social, and transportation; children’s questionnaires did not include occupational exposure questions) and gave a sample of hair from the back of their head as previously described [35]. During the second stage (i.e., 20–30 days following stage 1), a randomly-selected 30% of the participants completed the questionnaires again to assess reliability and the two questionnaires for each of these participants were randomly termed forms 1 and 2. All measurements were conducted at approximately the same time of the day and by the same trained investigators. For the completion of the questionnaires, a researcher read each question clearly and provided explanations to ensure that the participants understood what was requested. Thereafter, the participants selected one of the possible answers according to what they felt was true for them. The same procedure was followed for all questions, and no time limits were set for completing the questionnaires.

### SHS Questionnaires

The questionnaires that were initially developed for children and adults included a total of 48 and 64 questions, respectively, pertaining to all relevant sources of SHS exposure (i.e., SHS exposure at the home, workplace, public areas, and in the car), and contained both objective (e.g., how many members of your family smoke?) and subjective (e.g., how much do you think you are exposed to tobacco smoke at home?) questions. This approach was adopted to ensure that the questionnaires would capture both the time that someone spends with smokers (an objective proxy of SHS exposure) as well as his/her perceived exposure to SHS (a subjective proxy of SHS exposure) since individuals subjected to SHS for years may underestimate their exposure [32], [43]. The questions for each source of SHS (SHS exposure at the home, workplace, public areas, and in the car) are outlined in the Online Data Supplement (File S1).

### Hair Nicotine Analysis

A sample of hair 100–150 mg from the root at the back of the participants’ head was obtained as previously described [35]. Each hair sample was placed in a separate envelope and kept in a dry room at 22–25°C. When all hair samples were collected, they were washed to remove any external contamination and digested for 90 min at 60°C in 2 ml of 1 M NaOH. Hair samples extracted mechanically twice with 3 ml dichloromethane and the organic phases were separated, combined and evaporated to dryness. Analysis was performed using a liquid chromatography-mass spectrometry (Shimadzu LCMS–2010 EV) system equipped with an APCI interface. The column was a Discovery C18 HPLC column (25 cm×4.6 mm, 5 µm). Thirty µl from each extracted sample were entered in the column at temperature 45°C. A gradient of 50 mM ammonium acetate, pH = 5.1, (solvent A) and an acetonitrile (solvent B) were selected for routine use. The detection was done in SIM mode using ion fragments with m/z 163, 204 for nicotine and m/z 177, 218 for cotinine.

### Statistical Analysis

Three data analyses were conducted, each addressing one of the purposes of this study. The first data analysis aimed at developing questionnaires assessing SHS exposure in children and adult never-smokers. For this purpose, we conducted two principal factor analyses (one for children and one for adults) using the questionnaire data which included questions pertaining to all relevant sources of SHS exposure. The factor analyses were used to examine possible factor structures and identify specific items to create short versions of the questionnaires reflecting the main sources of SHS exposure. The suitability of the data for structure detection was assessed using the Kaiser-Meyer-Olkin Measure of Sampling Adequacy (KMO), indicating the proportion of variance in the variables that may be caused by underlying factors (>0.5 values suggest that the factor analysis results are useful), and Bartlett's test of sphericity, which tests the relationships between the variables and, hence, the suitability for structure detection (p<0.05 values suggest that the factor analysis results are useful). An eigenvalue >1 was used as an *a priori* criterion to determine the number of factors to be extracted from the data. Generally, factor loadings of r≥0.7 and r≤0.4 are considered high and low, respectively [44], [45]. In the factor analysis for children we excluded items that loaded with r<0.6 on any factor. In the factor analysis for adults we excluded items that loaded with r<0.7 on any factor since a large part of the variance was explained with factors of high loading (see Results section). For both analyses, items with weak loadings (i.e., r<0.4) and items that were highly correlated (indicating item redundancy) were discarded.

The aim of the second data analysis was to determine the validity of the children and adult questionnaires (short version) towards identifying individuals that experience increased SHS against hair nicotine. Two Receiver Operating Characteristics (ROC) curve analyses (one for children and one for adults) were applied to define cutoff points for the two short-version questionnaires using hair nicotine concentration as a reference standard. For this purpose, a positive diagnosis of high SHS exposure (SHS+) was assigned when hair nicotine was >0.87 and >0.42 ng/mg in children and adults, respectively, based on data collected from 31 countries [46]. The area under the ROC curve was estimated using the Delong non-parametric method [21], [47]. Calculated sensitivity and specificity with corresponding 95% confidence intervals (CI95%) were used to determine cutoff points that would allow a correct diagnosis for SHS+. Sensitivity was defined as the proportion of individuals diagnosed as SHS+ using the ROC results who demonstrated hair nicotine concentrations above the international health standards. Specificity was defined as the proportion of individuals diagnosed as disease free (i.e., SHS-) using the ROC results who demonstrated hair nicotine concentrations below the international health standards. Cohen’s Kappa statistic was used to evaluate the agreement between questionnaire diagnosis and the reference standard test (i.e., hair nicotine concentration).

The aim of the third data analysis was to assess the reliability of the children and adult short-version questionnaires using data from the randomly-selected 30% of the participants who completed them twice (20–30 days apart). For this purpose, the two questionnaires for each of these participants were randomly termed forms 1 and 2. As previously suggested [48], [49], reliability was assessed using correlation coefficients (Kendall’s Tau-b) and Wilcoxon signed ranks tests followed by 95% limits of agreement and percent coefficient of variation to quantify the amount of test-retest error. Finally, ROC curves were calculated using data from forms 1 and 2. Pair-wise differences in sensitivity and specificity at the cutoff point between forms 1 and 2 in SS-C and SS-A were examined with McNemar's Chi-square method [50] for matched, potentially correlated dichotomous (binary) tests. Pair-wise differences in the area under the ROC curve between forms 1 and 2 in SS-C and SS-A were examined with the non-parametric method developed by Zhou et al. [51]. Data were analyzed with SPSS (version 19, SPSS Inc., Chicago, Illinois) and NCSS 2007 (Number Cruncher Statistical Systems, Utah, USA) statistical software packages. The level of significance was set at p<0.05.

## Results

### Questionnaire Development (Analysis 1)

In children, the required factoring criteria were satisfied (KMO = 0.71; Bartlett’s test χ^2^ = 3553; p<0.001). Factor analysis of the initial 51 questions suggested that 3 factors explained 76% of the variance (factor loadings from each item appear in Table 1). The factors could also be seen as three subscales, with two items related to home SHS exposure (31% of variance), five items related to general SHS exposure (26% of variance), and two items related to social SHS exposure (19% of variance), as shown in Table 1. The final scale (short version of questionnaire) was termed Smoke Scale for Children (SS-C) and contained 9 items (see Table 1). The obtainable score range for the SS-C was 0–50.4, with higher numbers reflecting higher SHS exposure.

**Table 1:** Factor loadings for the Smoke Scale in children and adults.

CHILDREN					ADULTS				
Item	Question	Factors			Item	Question	Factors		
		1	2	3			1	2	3
1	How much do you think you are exposed totobacco smoke at home? (VAS)	−0.63	0.62		1	How many times per week do you usuallygo out to socialize?	0.89		
2	How many members of your family smokeinside your home?	−0.60	0.63		2	When you go out to bars, how many hoursdo you usually stay?	0.75		
3	How much do you think you are exposed totobacco smoke when you go out to socialize?(Likert)		0.80		3	How many times per week do you usuallygo out to coffee shops?	0.74		
4	How many times per week do you usuallygo out to socialize?		0.68		4	When you go out to coffee shops, how manyhours do you usually stay?	0.74		
5	How much do you think you are exposed totobacco smoke at home? (Likert)		0.67		5	How many times per week do you usuallygo out to bars?	0.74		
6	When you go out to coffee shops, how manyhours do you usually stay?		0.62		6	How many cigarettes does each of yoursmoker co-workers usually smoke at workper day?		0.78	
7	How many times per week do you usuallygo out to bars?			−0.61	7	How much do you think you are exposedto tobacco smoke at work? (VAS)		0.77	
8	How many people smoke inside the bars youusually go to?			−0.60	8	At work, how many hours per day do youusually spend with smoker co-workers?		0.70	
9	How many people smoke inside thetaverns/restaurants you usually go to?			0.60	9	How much do you think you are exposedto tobacco smoke at home? (Likert)			0.74

Note: VAS = 10 cm visual analogue scale; Likert = 5-level likert scale (not at all, somewhat, moderately, a lot, extremely).

In adults, the required factoring criteria were satisfied (KMO = 0.67; Bartlett’s test χ^2^ = 2829; p<0.001). Factor analysis of the initial 67 questions suggested that 3 factors explained 71% of the variance (factor loadings from each item appear in Table 1). The factors could also be seen as three subscales, with five items related to social SHS exposure (32% of variance), three items related to occupational SHS exposure (24% of variance), and one item related to home SHS exposure (15% of variance), as shown in Table 1. The final scale (short version of questionnaire) was termed Smoke Scale for Adults (SS-A) and contained nine items (see Table 1). The obtainable score range for the SS-A was 0–49.8, with higher numbers reflecting higher SHS exposure.

### Validity Assessment (Analysis 2)

**Figure 1:**
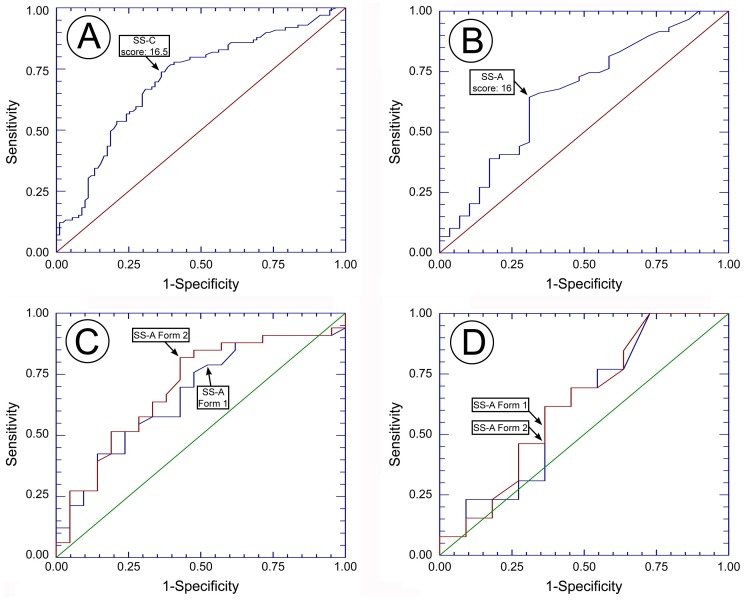
Receiver operating characteristics (ROC) curves for SS-C (A) and SS-A (B) indicating the designated cut off points at 16.5 and 16, respectively. The points at the ROC curve at the designated cut offs for SS-C (C) and SS-A (D) forms 1 and 2 are also illustrated.

The results for hair nicotine and the prevalence rates for SHS+ and SHS- in children and adult never-smokers appear in Table 2. Using the hair nicotine results, chi-square demonstrated that the prevalence of SHS+ in children (52.1±7.1%) was significantly lower than in adults (67.1±9.8%) (χ^2^ = 5.47, p = 0.019). ROC curve analyses revealed that the most appropriate cutoffs for SHS+ in SS-C and SS-A were “16.5” and “16”, respectively. Relevant univariate statistics and ROC curve analyses for the designated cutoffs appear in Table 3 and Figure 1:A and B. Hair nicotine analysis suggested that 99 children and 59 adults were SHS+. The SS-C and the SS-A diagnosed 73 children and 38 adults as SHS+, respectively. Cohen’s Kappa statistic demonstrated significant agreement with the hair nicotine measurement for both the SS-C (z = 5.22, p<0.001) and the SS-A (z = 2.961, p = 0.003).

**Table 2:** Results (median ± interquartile range) for hair nicotine and prevalence rates (±95% confidence interval) for SHS+ and SHS- in children and adult never-smokers.

		Hair nicotine(ng/mg)	SHS+	SHS–
Children	Sample	0.92±2.69	0.52±0.07†	0.48±0.07†
	Boys	0.93±3.19	0.52±0.11	0.48±0.11
	Girls	0.90±2.20	0.52±0.09	0.48±0.09
Adults	Sample	0.77±1.70	0.67±0.10*†	0.33±0.10*†
	Men	1.20±3.11	0.70±0.20*	0.30±0.20*
	Women	0.74±1.66	0.66±0.11*	0.34±0.11*

Note: * = χ^2^ significant difference (p<0.05) between SHS+ and SHS–.

= χ^2^ significant difference (p<0.05) between children and adults.

Key: SHS+ = positive diagnosis of SHS exposure using the forms; SHS– = negative diagnosis of SHS exposure using the forms.

**Table 3:** Results for ROC curve and McNemar Chi-Square analyses for the designated cutoffs for SHS+ in SS-C and SS-A.

	SE±CI95%	SP±CI95%	PPV±CI95%	NPV±CI95%	LR±CI95%	AUC±SE
SS-C	0.74±0.09	0.64±0.10	0.69±0.09	0.69±0.01	2.03±0.11	0.71±0.04*
SS-A	0.64±0.12	0.69±0.17	0.81±0.11	0.49±0.15	2.08±0.21	0.66±0.06*

Note: * = AUC test statistically significant (p<0.05) from 0.5 (i.e., no diagnostic ability).

Key: ROC = receiver operating characteristics; SE = sensitivity; SP = specificity; PPV = positive predicted value; NPV = negative predicted value; LR = likelihood ratio; AUC = area under the ROC curve; CI95% = 95% confidence interval; SE = standard error.

### Reliability Assessment (Analysis 3)

The reliability results in children are presented in Table 4 and Figure 1:C. The SS-C scores of forms 1 and 2 were highly correlated (tau-b = 0.90, p<0.001), and a Wilcoxon signed ranks test demonstrated no statistically significant differences between them [z = −1.85, p = 0.07]. The 95% limits of agreement indicated that a score of 20 on one day can be as high as 23 or as low as 18.84 on another day. The calculated ROC curves in SS-C demonstrated similar results at the cutoff of 16.5. Specifically, comparisons of sensitivities and specificities for each form with McNemar's chi square test for binary tests and matched data demonstrated no statistically significant differences (p>0.05). Pair-wise comparison in the area under the ROC curve between forms 1 and 2 demonstrated no statistically significant differences (z = −1.4, p = 0.16).

**Table 4:** Reliability results for SS-C and SS-A.

	Form	Median±IR	95% LoA	%CV	SE±CI95%	SP±CI95%	PPV±CI95%	NPV±CI95%	AUC±SE
SS-C	1	17.90±10.80	0.9±2.08	5.23	0.79±0.15	0.48±0.20	0.70±0.15	0.59±0.22	0.67±0.08*
	2	18.80±9.30			0.81±0.16	0.57±0.22	0.75±0.14	0.67±0.20	0.69±0.08*
SS-A	1	18.73±11.72	0.8±2.78	7.94	0.54±0.27	0.64±0.28	0.64±0.28	0.54±0.27	0.62±0.12*
	2	17.60±11.55			0.46±0.27	0.64±0.28	0.60±0.30	0.50±0.26	0.63±0.12*

Note: * = AUC test statistically significant (p<0.05) from 0.5 (i.e., no diagnostic ability).

Key: IR = interquartile range; 95%LoA = 95% limits of agreement; %CV = percent coefficient of variation; SE = sensitivity; SP = specificity; PPV = positive predicted value; NPV = negative predicted value; LR = likelihood ratio; AUC = area under the ROC curve; CI95% = 95% confidence interval; SE = standard error.

The reliability results in adults are presented in Table 4 and Figure 1:D. The SS-A scores of forms 1 and 2 were highly correlated (tau-b = 0.96, p<0.001), and a Wilcoxon signed ranks test demonstrated no statistically significant differences between them [z = −1.88, p = 0.06]. The 95% limits of agreement indicated that a score of 20 on one day can be as high as 23.6 or as low as 18.1 on another day. The calculated ROC curves in SS-A demonstrated similar results at the cutoff of 16. Specifically, comparisons of sensitivities and specificities for each form with McNemar's chi square test for binary tests and matched data demonstrated no statistically significant differences (p>0.05). Pair-wise comparison in the area under the ROC curve between forms 1 and 2 demonstrated no statistically significant differences (z = −0.45, p = 0.652).

## Discussion

Since hair nicotine concentration can provide an accurate assessment of SHS exposure for the preceding three months in both children and adults [34]–[39], our goal was to develop questionnaires able to identify children and adult never-smokers that experience increased SHS as well as to determine their validity and reliability against hair nicotine. To construct the questionnaires, we asked a series of 51 and 67 questions in children and adults, respectively, pertaining to all relevant sources of SHS exposure (i.e., SHS exposure at the home, workplace, public areas, and in the car) and including both objective (e.g., how many members of your family smoke?) and subjective (e.g., how much do you think you are exposed to tobacco smoke at home?) questions. We adopted this approach to ensure that the questionnaires would capture both the time that someone spends with smokers (an objective proxy of SHS exposure) as well as his/her perceived exposure to SHS (a subjective proxy of SHS exposure) since individuals subjected to SHS for years may underestimate their exposure [32], [43]. Factor analysis was used, thereafter, to examine possible factor structures and identify specific questions to create short versions of the questionnaires reflecting the main sources of SHS exposure. We adopted this methodology instead of conducting factor analyses only on the items that were moderately/highly correlated with hair nicotine concentration because, based on relevant guidelines [45], our goal was to reduce the data while retaining as much of the original measures’ total variance as possible. The value of this approach was confirmed when we attempted to construct brief questionnaires by selecting items that were believed to provide accurate SHS+ diagnosis. For instance, an attempt to use the visual analogue scale items pertaining to the perceived exposure to SHS at home, the workplace, and when socializing resulted reduced sensitivity in children and no significant agreement with hair nicotine in adults. In this light, the resulting SS-C and SS-A questionnaires are the first to accurately reflect the true exposure of an individual to SHS, and not just that occurring during the past few days.

Our factor analysis demonstrated that social SHS exposure is most prevalent in children, followed closely by home SHS exposure. Indeed, the resulting Smoke Scale for Children (SS-C) included six questions pertaining to social SHS exposure (explaining ∼40% of the variance) and three questions pertaining to home SHS exposure (explaining ∼36% of the variance). These results are in line with the results of the Global Youth Tobacco Survey [52] conducted in 151 countries and territories during 2000–2007 which showed that 42.5% of never smokers were exposed to SHS at home and 55.1% were exposed in public places. These results together with the SHS exposure conditions for nonsmokers in Greece at the time the data were collected (as described above) confirm that our sample is representative of the vast majority of children never-smokers across the world.

Factor analysis of the initial 67 questions in adults revealed that social SHS exposure is most prevalent in this subgroup, followed by occupational and home SHS exposure. The resulting Smoke Scale for Adults (SS-A) included five questions pertaining to social exposure (explaining 32% of the variance), three questions pertaining to occupational SHS exposure (explaining 24% of the variance), and one question pertaining to home SHS exposure (explaining 15% of the variance). These results are in reasonable agreement with the results of the Global Adults Tobacco Survey [53] conducted in 17 countries since 2007 which has shown that 45.2% of never smokers are exposed to SHS in public places, 44.5% are exposed to SHS at home, and 37.7% are exposed in the workplace. Thus, it is reasonable to assume that our sample is representative of the vast majority of adult never-smokers across the world.

The English versions of the SS-C and the SS-A, their scoring manuals, and the adopted translation methodology have been included as Online Data Supplements (Files S1, S2, and S3). The total score of all questions in each questionnaire was used to establish positivity criteria for children and adults using hair nicotine concentration as a reference standard and international exposure limits (i.e., >0.87 and >0.42 ng/mg hair nicotine in children and adults, respectively) of ∼2500 nonsmoking children and adults in 31 countries [46] for a positive diagnosis of high SHS exposure (SHS+). While adopting different cutoffs would have affected the validity of SS-C and SS-A, we selected these cutoffs as they were proposed by the most wide-spread study to be published during the past decade. The optimal cutoff point in establishing each positivity criterion was determined by selecting a score that retained a low frequency of both, false-positive and false-negative, rates. This conservative approach is considered appropriate since SHS+ is a serious health threat but it does not require immediate medical attention. Further, this approach in selecting positivity criteria limits both needless lifestyle modifications as a form of intervention (false-positive) and harmful SHS exposure (false-negative) for an individual that would have, otherwise, benefited from an early SHS+ diagnosis.

The ROC curve analyses demonstrated that children and adults reporting SS-C and SS-A scores above 16.5 and 16, respectively, are considered to have a high probability for SHS+ and should be referred to a health specialist for further testing. The validity results for SS-C and SS-A demonstrated high sensitivity and specificity as well as significant agreement with hair nicotine concentration. Furthermore, both forms showed adequate reliability. Despite capturing some individuals with high scores for reasons other than SHS, the SS-C and SS-A appear to identify the majority of SHS+ individuals demonstrating significant agreement with the reference standard hair nicotine concentration. According to the sensitivity and specificity obtained, in a hypothetical population of 2000 equally distributed children and adults, the SS-C and SS-A would identify correctly 385/520 and 429/670 individuals, respectively. Therefore, more than 2 out of 3 children and adults who are exposed to SHS above safety limits can be detected simply by answering a set of nine questions. Moreover, hair nicotine analysis would be required to confirm a diagnosis of SHS+ in 809 individuals of the total 2000. As the total cost of screening such a large population using hair nicotine would reach a minimum of €46,000 [54], the use of the SS-C and SS-A would reduce the total cost for SHS+ screening by 60%.

The SS-C and SS-A have been standardized against hair nicotine, a biomarker that has shown an improved capacity to discriminate SHS exposure status compared to urinary cotinine [34], [55]. While the validity of hair nicotine to detect levels of chronic SHS exposure has been repeatedly demonstrated in both children and adults [32]–[39], it is important to note that this method does not allow for the determination of nicotine sources; that is, nicotine directly deposited on the hair from the environment and nicotine incorporated in the hair through SHS. Hair treatment methods such as preliminary washing (adopted in the current study) can remove the majority of the nicotine deposited on the hair exterior. However, some residual nicotine remains, causing external contamination [56]. In our study, we feel that this external contamination was minimal, since all our subjects were never-smokers.

Future research should aim to further increase the validity of the SS-C and, primarily, the SS-A. It is important to note that 27% of the children and 36% of the adults with SHS+ will not be detected using the SS-C and SS-A, respectively. This limits the degree to which intervention programs would be made available to these individuals. However, hair nicotine analysis is neither practical nor cost-effective. It requires trained staff, specialized equipment, and cost approximately €23 per sample [54]. Furthermore, since testing can be conducted only on an individual basis, it is not possible to conduct mass screenings over short time periods. As a result, individuals and/or organizations often object to time consuming screenings due to interference with daily routines. Hence, it seems reasonable to assume that screening procedures are not available to the majority of the populations around the world due to financial and logistic reasons. The SS-C and SS-A allow for mass screenings that can be conducted in very short periods of time with a minimum budget. Therefore, despite the missed screening of a small number of individuals, the majority of SHS+ children and adults, who would otherwise escape screening, receive referral to a health specialist. Based on the evidence presented herein, we conclude that the SS-C and the SS-A represent valid, reliable, practical, and inexpensive instruments to identify children and adult never-smokers exposed increased SHS.

## Supporting Information

File S1 **Online Data Supplement.** A supplement containing the questions for each source of SHS (SHS exposure at the home, workplace, public areas, and in the car), the adopted translation methodology, as well as the scoring manuals for the SS-C and the SS-A.
(DOCX)

File S2 **Smoke Scale for Children Questionnaire.** The English version of the SS-C questionnaire.
(DOCX)

File S3 **Smoke Scale for Adults Questionnaire.** The English version of the SS-A questionnaire.
(DOCX)
