# Supplementary material for: Instruments to Assess Secondhand Smoke Exposure in Large Cohorts of Never Smokers: The Smoke Scales
Source: PLoS One. 2014 Jan 21;9(1):e85809. doi: 10.1371/journal.pone.0085809 (PMC3897519; doi:10.1371/journal.pone.0085809)
Supplement: File S1 — Online Data Supplement. A supplement containing the questions for each source of SHS (SHS exposure at the home, workplace, public areas, and in the car), the adopted translation methodology, as well as the scoring manuals for the SS-C and the SS-A. (DOCX) [file pone.0085809.s001.docx]

**INSTRUMENTS TO ASSESS SECONDHAND SMOKE EXPOSURE IN LARGE COHORTS: THE SMOKE SCALES**

Maria Misailidi, Manolis N. Tzatzarakis, Mathaios P. Kavvalakis, Yiannis Koutedakis, Aristidis M. Tsatsakis, Andreas D. Flouris

**ONLINE DATA SUPPLEMENT**

**ORIGINAL SHS QUESTIONNAIRES**

The questionnaires that were initially developed for children and adults included a total of 48 and 64 questions, respectively, pertaining to all relevant sources of SHS exposure, and included both objective (e.g., how many members of your family smoke?) and subjective (e.g., how much do you think you are exposed to tobacco smoke at home?) questions. This approach was adopted to ensure that the questionnaires would capture both the time that someone spends with smokers (an objective proxy of SHS exposure) as well as his/her perceived exposure to SHS (a subjective proxy of SHS exposure) since individuals subjected to SHS for years may underestimate their exposure [[1](#_ENREF_1),[2](#_ENREF_2)]. The questions for each source of SHS are outlined below:

Home exposure

A total of 31 questions were included in both the children and adult questionnaires: “Is smoking allowed inside your home?”; “Excluding family members, how many smokers usually visit your home every week?”; “How many members of your family smoke?”; “How many rooms does your home have?”; “How many members of your family smoke inside your home?”; “On average, how many cigarettes per day does each family member smoke?” (individual questions for up to 6 family members); “In which rooms does each family member smoke?” (individual questions for up to 6 family members); “Which part(s) of the day does each family member smoke inside your home (select one or more: morning, noon, afternoon, evening)?” (individual questions for up to 6 family members); “How much do you think you are exposed to tobacco smoke at home?” (select one: not at all, somewhat, moderately, a lot, extremely); “How much do you think you are exposed to tobacco smoke at home?” (10 cm visual analogue scale).

Occupational exposure

A total of 16 questions were included only in the adult questionnaire: “Did you work during the past three months?” “Is smoking allowed at your workplace?”; “Describe your workplace (select one: outdoors, semi-outdoors, indoors)?”; “Excluding co-workers, how many smokers usually visit your workplace every week?”; “How many of your co-workers smoke inside your workplace?”; “How many cigarettes per day does each of your co-workers usually smoke inside the workplace?” (individual questions for co-workers smoking 1, 1.5-2, and >2 cigarette packs per day); “At work, how many hours per day do you usually spend with smoker co-workers?” “How much do you think you are exposed to tobacco smoke at your workplace?” (select one: not at all, somewhat, moderately, a lot, extremely); “How much do you think you are exposed to tobacco smoke at your workplace?” (10 cm visual analogue scale).

Social exposure

A total of 15 questions were included in both the children and adult questionnaires: “How many times per week do you usually go out to socialize?”; “How many times per week do you usually go out to friends’ houses?”; “How many people smoke inside the friends’ houses you usually go to?” (select one: no one, some, half, a lot, everyone); “When you go out to friends’ houses, how many hours do you usually stay?”; “How many times per week do you usually go out to taverns/restaurants?”; “How many people smoke inside the taverns/restaurants you usually go to?” (select one: no one, some, half, a lot, everyone); “When you go out to taverns/restaurants, how many hours do you usually stay?”; “How many times per week do you usually go out to coffee shops?”; “How many people smoke inside the coffee shops you usually go to?” (select one: no one, some, half, a lot, everyone); “When you go out to coffee shops, how many hours do you usually stay?”; “How many times per week do you usually go out to bars?”; “How many people smoke inside the bars you usually go to?” (select one: no one, some, half, a lot, everyone); “When you go out to bars, how many hours do you usually stay?”; “How much do you think you are exposed to tobacco smoke when you go out to socialize?” (select one: not at all, somewhat, moderately, a lot, extremely); “How much do you think you are exposed to tobacco smoke when you go out to socialize?” (10 cm visual analogue scale).

Transportation exposure

A total of 2 questions were included in both the children and adult questionnaires: “Is smoking allowed inside the car that you mostly use?”, and “On average, how many hours per day do you spend inside this car?”.

**QUESTIONNAIRE TRANSLATION AND LINGUISTIC VALIDATION**

**Methodology**

Since the original SS-C and SS-A questionnaires were prepared in Greek, appropriate procedures were followed to translate them in English. Specifically, the translation to English was made using a forward-backward translation protocol according to appropriate guidelines [[3](#_ENREF_3),[4](#_ENREF_4)]. The Greek versions of the SS-C and SS-A were translated to English by two independent translators (ADF and AMT). Inconsistencies between the different versions created by the translators were resolved via consensus leading to one draft English version for the SS-C and the SS-A. Subsequently, these draft questionnaires were translated back to Greek by two independent translators (MNT and MPK) who were not involved in creating the first English drafts. The resulting drafts in Greek were compared with the original SS-C and SS-A by an independent committee, who reported that no significant linguistic differences were observed between the questionnaires. Thereafter, the Greek and English versions of the SS-C and SS-A were completed by 16 children (8 boys, 8 girls) and 16 adults (8 men, 8 women), respectively, who were fluent in both languages.

**Statistical Analyses**

As previously suggested [[5](#_ENREF_5),[6](#_ENREF_6)], reliability was assessed using correlation coefficients (Kendall’s Tau-b) and Wilcoxon signed ranks tests followed by 95% limits of agreement and percent coefficient of variation to quantify the amount of test-retest error. Data were analyzed with SPSS (version 19, SPSS Inc., Chicago, Illinois). The level of significance was set at p<0.05.

**Results**

The reliability results in children are presented in Table S1. The SS-C scores of the Greek and English versions were highly correlated (tau-b=0.98, p<0.001), and a Wilcoxon signed ranks test demonstrated no statistically significant differences between them [z=-0.24, p=0.81]. The 95% limits of agreement indicated that a score of 20 on one language can be as high as 20.62 or as low as 19.39 on another language. The SS-A scores of the Greek and English versions were highly correlated (tau-b=0.98, p<0.001), and a Wilcoxon signed ranks test demonstrated no statistically significant differences between them [z=-1.50, p=0.13]. The 95% limits of agreement indicated that a score of 20 on one language can be as high as 20.55 or as low as 19.16 on another language.

**Conclusions**

Based on the above results, it is concluded that the English versions of the SS-C and the SS-A were valid and reflected effectively the results of the Greek versions that were extensively validated in this study. The English versions of the SS-C and the SS-A and their scoring manuals are included below.

**REFERENCES**

1. Carrillo AE, Metsios GS, Flouris AD (2009) Effects of secondhand smoke on thyroid function. Inflamm Allergy Drug Targets 8.

2. Pichini S, Garcia-Algar O, Munoz L, Vall O, Pacifici R, et al. (2003) Assessment of chronic exposure to cigarette smoke and its change during pregnancy by segmental analysis of maternal hair nicotine. J Expo Anal Environ Epidemiol 13: 144-151.

3. Guillemin F, Bombardier C, Beaton D (1993) Cross-cultural adaptation of health-related quality of life measures: literature review and proposed guidelines. J Clin Epidemiol 46: 1417-1432.

4. Guillemin F (1995) Cross-cultural adaptation and validation of health status measures. Scand J Rheumatol 24: 61-63.

5. Flouris AD, Koutedakis Y, Nevill A, Metsios GS, Tsiotra G, et al. (2004) Enhancing specificity in proxy-design for the assessment of bioenergetics. J Sci Med Sport 7: 197-204.

6. Flouris AD, Metsios GS, Koutedakis Y (2005) Enhancing the efficacy of the 20 m multistage shuttle run test. Br J Sports Med 39: 166-170.

| **Table S1.** Results for the translation and linguistic validation of the SS-C and SS-A. | | | | |
| --- | --- | --- | --- | --- |
|  | Language | Median±IR | 95% LoA | %CV |
| SS-C | Greek | 22.20±12.27 | 0.01±0.61 | 1.49 |
|  | English | 22.10±12.20 |  |  |
| SS-A | Greek | 11.70±15.83 | -0.14±0.70 | 2.10 |
|  | English | 11.65±15.40 |  |  |
| Key: IR=interquartile range; 95%LoA=95% limits of agreement; %CV=percent coefficient of variation. | | | | |

**SCORING MANUAL FOR THE SS-C**

Please, take into account the scoring instructions below to calculate the sum for the SS-C questionnaire:

**Questions 1-4.** Use the number indicated by the participant.

**Questions 5-8.** The scores for each selection are:

Not at all = 1

Somewhat = 2

Moderately = 3

A lot = 4

Extremely = 5

**Question 9.** The score in this Visual Analogue Scale is determined by measuring in cm from the left hand end of the line to the point that the participant marks.

**SCORING MANUAL FOR THE SS-A**

Please, take into account the scoring instructions below to calculate the sum for the SS-A questionnaire:

**Questions 1-7.** Use the number indicated by the participant.

**Question 8.** The scores for each selection are:

Not at all = 1

Somewhat = 2

Moderately = 3

A lot = 4

Extremely = 5

**Question 9.** The score in this Visual Analogue Scale is determined by measuring in cm from the left hand end of the line to the point that the participant marks.
