# Supplementary material for: Instruments to Assess Secondhand Smoke Exposure in Large Cohorts of Never Smokers: The Smoke Scales
Source: PLoS One. 2014 Jan 21;9(1):e85809. doi: 10.1371/journal.pone.0085809 (PMC3897519; doi:10.1371/journal.pone.0085809)
Supplement: File S2 — Smoke Scale for Children Questionnaire. The English version of the SS-C questionnaire. (DOCX) [file pone.0085809.s002.docx]

**SMOKE SCALE**

**CHILDREN VERSION**

Name: Birth date: ____ / ____ / ____ Sex: M / F

MM DD YY

Grade:

**INSTRUCTIONS:**

PLEASE RESPECT YOUR FELLOW STUDENTS’ PRIVACY BY KEEPING YOUR EYES ON YOUR OWN PAPER! This survey evaluates how much you are exposed to second hand tobacco smoke. You will have to read a sentence and then give the answer you think is ***MOST LIKE YOU***. In the first part of the survey your response will be a number, in the second part of the survey you will be asked to circle the answer that is the closest to how you feel, and in the third part of the survey you will have to draw a vertical line through a scale.

What is second hand tobacco smoke: the smoke you inhale when people near you use tobacco products.

Try the following examples:

**SAMPLE QUESTION 1. Answer with a number:**

**How many members of your family go to school? _____**

Once you decide, fill in your answer within the space provided.

That shouldn’t be too hard for you to decide! Once you decide, just circle the answer that is most like you.

**SAMPLE QUESTION 2. Circle the answer closest to how you feel:**

**How much do you like math? Not at all Somewhat Moderately A lot Extremely**

Simply draw a vertical line through the scale indicating how much you like playing football. The line should be in reference to “Not at all” appearing at the left end of the scale, and “Very much” appearing at the right end of the scale.

**SAMPLE QUESTION 3. Draw a vertical line through the scale below:**

**Do you like playing football?**

REMEMBER: THERE ARE NO CORRECT OR INCORRECT ANSWERS, JUST WHAT IS ***MOST LIKE YOU***.

Now you are ready to start filling in this form. Take your time and do the whole form carefully. If you have any questions just ask! If you think you are ready you can start now.

| **PART 1: Answer with a number** | | | | | | | |
| --- | --- | --- | --- | --- | --- | --- | --- |
| **Question 1:** | How many members of your family smoke inside your home? _____ | | | | | | |
| **Question 2:** | How many times per week do you usually go out to socialize? _____ | | | | | | |
| **Question 3:** | When you go out to coffee shops, how many hours do you usually stay? _____ | | | | | | |
| **Question 4:** | How many times per week do you usually go out to bars? _____ | | | | | | |
| **PART 2: Circle the answer closest to how you feel** | | | | | | | |
| **Question 5:** | How much do you think you are exposed to second hand tobacco smoke at home? | | | | | | |
|  | Not at all | Somewhat | | Moderately | A lot | | Extremely |
| **Question 6:** | How much do you think you are exposed to second hand tobacco smoke when you go out to socialize? | | | | | | |
|  | Not at all | Somewhat | | Moderately | A lot | | Extremely |
| **Question 7:** | How many people smoke inside the taverns/restaurants you usually go to? | | | | | | |
|  | No one | Some | | Half | A lot | | Everyone |
| **Question 8:** | How many people smoke inside the bars you usually go to? | | | | | | |
|  | No one | Some | | Half | A lot | | Everyone |
| **PART 3: Draw a vertical line through the scales below:** | | | | | | | |
| **Question 9:** | How much do you think you are exposed to second hand tobacco smoke at home? | | | | | | |
|  | 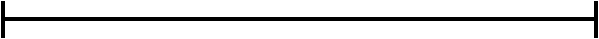 | | | | | | |
|  | Not at all | |  | | | Very much | |

**THANK YOU VERY MUCH FOR COMPLETING THE SMOKE SCALE FOR CHILDREN! ☺**
