# Supplementary material for: Instruments to Assess Secondhand Smoke Exposure in Large Cohorts of Never Smokers: The Smoke Scales
Source: PLoS One. 2014 Jan 21;9(1):e85809. doi: 10.1371/journal.pone.0085809 (PMC3897519; doi:10.1371/journal.pone.0085809)
Supplement: File S3 — Smoke Scale for Adults Questionnaire. The English version of the SS-A questionnaire. (DOCX) [file pone.0085809.s003.docx]

**SMOKE SCALE**

**ADULTS VERSION**

Name: Birth date: ____ / ____ / ____ Sex: M / F

MM DD YY

**INSTRUCTIONS:**

This survey evaluates how much you are exposed to second hand smoke. You will have to read a sentence and then give the answer you think is ***MOST LIKE YOU***. In the first part of the survey your response will be a number, in the second part of the survey you will be asked to circle the answer that is the closest to how you feel, and in the third part of the survey you will have to draw a vertical line through a scale.

What is second hand tobacco smoke: the smoke you inhale when people near you use tobacco products.

Try the following examples:

**SAMPLE QUESTION 1. Answer with a number:**

**How many coffees do you normally drink per day? _____**

Once you decide, fill in your answer within the space provided.

Once you decide, just circle the answer that is most like you.

**SAMPLE QUESTION 2. Circle the answer closest to how you feel:**

**How much do you like exercise? Not at all Somewhat Moderately A lot Extremely**

**SAMPLE QUESTION 3. Draw a vertical line through the scale below:**

**Do you like watching football?**

Simply draw a vertical line through the scale indicating how much you like watching football. The line should be in reference to “Not at all” appearing at the left end of the scale, and “Very much” appearing at the right end of the scale.

REMEMBER: THERE ARE NO CORRECT OR INCORRECT ANSWERS, JUST WHAT IS ***MOST LIKE YOU***.

Now you are ready to start filling in this form. Take your time and do the whole form carefully. If you have any questions just ask the examiner or contact FAME Lab researchers at www.famelab.gr! If you think you are ready you can start now.

| **PART 1: Answer with a number** | | | | | | | |  |
| --- | --- | --- | --- | --- | --- | --- | --- | --- |
| **Question 1:** | At work, how many hours per day do you usually spend with smoker co-workers? _____ | | | | | | |  |
| **Question 2:** | How many cigarettes/day does each of your co-workers usually smoke inside the workplace? _____ | | | | | | | |
| **Question 3:** | How many times per week do you usually go out to socialize? _____ | | | | | | |  |
| **Question 4:** | How many times per week do you usually go out to coffee shops? _____ | | | | | | |  |
| **Question 5:** | When you go out to coffee shops, how many hours do you usually stay? _____ | | | | | | |  |
| **Question 6:** | How many times per week do you usually go out to bars? _____ | | | | | | |  |
| **Question 7:** | When you go out to bars, how many hours do you usually stay? _____ | | | | | | |  |
| **PART 2: Circle the answer closest to how you feel** | | | | | | | |  |
| **Question 8:** | How much do you think you are exposed to second hand tobacco smoke at home? | | | | | | |  |
|  | Not at all | Somewhat | | Moderately | A lot | | Extremely |  |
| **PART 3: Draw a vertical line through the scale below:** | | | | | | | |  |
| **Question 9:** | How much do you think you are exposed to second hand tobacco smoke at work? | | | | | | |  |
|  | 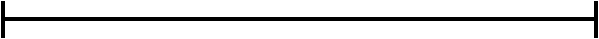 | | | | | | |  |
|  | Not at all | |  | | | Very much | |  |

**THANK YOU VERY MUCH FOR COMPLETING THE SMOKE SCALE FOR ADULTS! ☺**
